# Supplementary material for: Reliability Associated with the Measurement of Continuous Variables in Veterinary Medicine: What the Different Possible Indicators Tell, and How to Use and Report Them
Source: Animals (Basel). 2023 Sep 2;13(17):2793. doi: 10.3390/ani13172793 (PMC10486732; doi:10.3390/ani13172793)
Supplement: Supplementary file 1 [file animals-13-02793-s001.zip › animals-2564222-supplementary.pdf]

Supplementary Materials

Reliability Associated with the Measurement of Continuous Variables in Veterinary Medicine: What the Different Possible Indicators Tell, and How to Use and Report Them

Sébastien Buczinski

Département des Sciences Cliniques, Faculté de Médecine Vétérinaire, Université de Montréal, St-Hyacinthe, QC J2S 2M2, Canada; s.buczinski@umontreal.ca; Tel.: +1-450-773-8521 (ext. 8675)

Contents

A. DATASET USED FOR ILLUSTRATION PURPOSE: FEEDLOT LUNG CONSOLIDATION DEPTH BY 2 RATERS **2**

B. PEARSON AND SPEARMAN CORRELATION: FIGURE 1 **5**

C. DEMING AND PASSING BABLOK REGRESSION: FIGURE 2 **7**

D. AGREEMENT PLOT **8**

D.1 Agreement plot (default Bland-Altman method): FIGURE 3 8

D.2 Adjusted agreement plot allowing differential and proportional bias : FIGURE 4 9

libraries required

```
library(tidyverse)
library(blandr)
library(ggpubr)
library(ggExtra)
library(datapasta)# reprex used for creating and pasting the dataset
library(MethodCompare)#Bland-Altman with proportional bias
library(mcr)#passing bablok et deming regression
```

This is the supplementary file from the article :Reliability associated with measurement of continuous variables in veterinary medicine: what tell the different possible indicators and how to use and report them properly?.

A. DATASET USED FOR ILLUSTRATION PURPOSE: FEEDLOT LUNG CONSOLIDATION DEPTH BY 2 RATERS

The data used are a subset of data reported in a recent paper reporting reliability of raters assessing lug consolidation depth in scoring the same 50 videoloops of feedlot calves See **Buczinski et al., Inter-rater agreement and reliability of thoracic ultrasonographic findings in feedlot calves, with or without naturally occurring bronchopneumonia. J Vet. Intern. Med. 2018 Sep;32(5):1787-1792. doi: 10.1111/jvim.15257**. The dataset is online. For illustration purpose I have used the table between rater 1 (*Depth_1*) and rater 2 (*Depth_1*) assessing lung consolidation depth in cm. A total of 50 rows and 2 columns are obtained in a `rel` table with `Rater_1`, and `Rater_2` variables respectively.

```
rel <- tibble::tribble(
  ~Rater_1, ~Rater_2,
         7,        0,
         0,        0,
       0.5,      0.5,
         3,        3,
         5,        4,
         7,      6.5,
         6,        5,
         2,        0,
         3,        3,
         0,        0,
         3,        3,
         3,        2,
         6,        4,
         0,        0,
         6,        3,
         2,        0,
         0,        0,
         0,        0,
         4,      4.5,
         1,        1,
         0,        0,
         3,        3,
         0,        0,
         2,        2,
         4,        3,
         0,        0,
         4,        3,
         4,      4.5,
         0,        0,
         0,        0,
         3,        2,
         2,        2,
         4,        4,
         0,        0,
         0,        0,
```

```
        0,        0,
        0,      0.5,
        3,        2,
        0,        0,
      0.5,      0.5,
        0,        0,
        3,        3,
        0,        0,
        1,      0.5,
        0,        0,
        0,        0,
        3,        3,
        3,        3,
        2,      1.5,
        0,        0
  )

# I'll add for illustration purpose an error term (mean 0, sd=0.2) to avoid overlaying of dots with 0,0
set.seed(1234) # reproducibility seed
A=abs(rnorm(nrow(rel),mean=0,sd=.2))
rel$Rater_1=rel$Rater_1+A
set.seed(12345) # reproducibility seed
B=abs(rnorm(nrow(rel),mean=0,sd=.2))
rel$Rater_2=rel$Rater_2+B
```

We can look at the data at glance

```
# Save the scatter plot in a variable
p <- ggplot(rel, aes(x = Rater_1,  y = Rater_2)) +
  geom_point(color="black")+theme_classic()+
  labs(x="Operator_1", y="Operator_2")
# Plot the scatter plot with marginal histograms
ggMarginal(p, type = "histogram", fill="red",alpha=0.4)
```

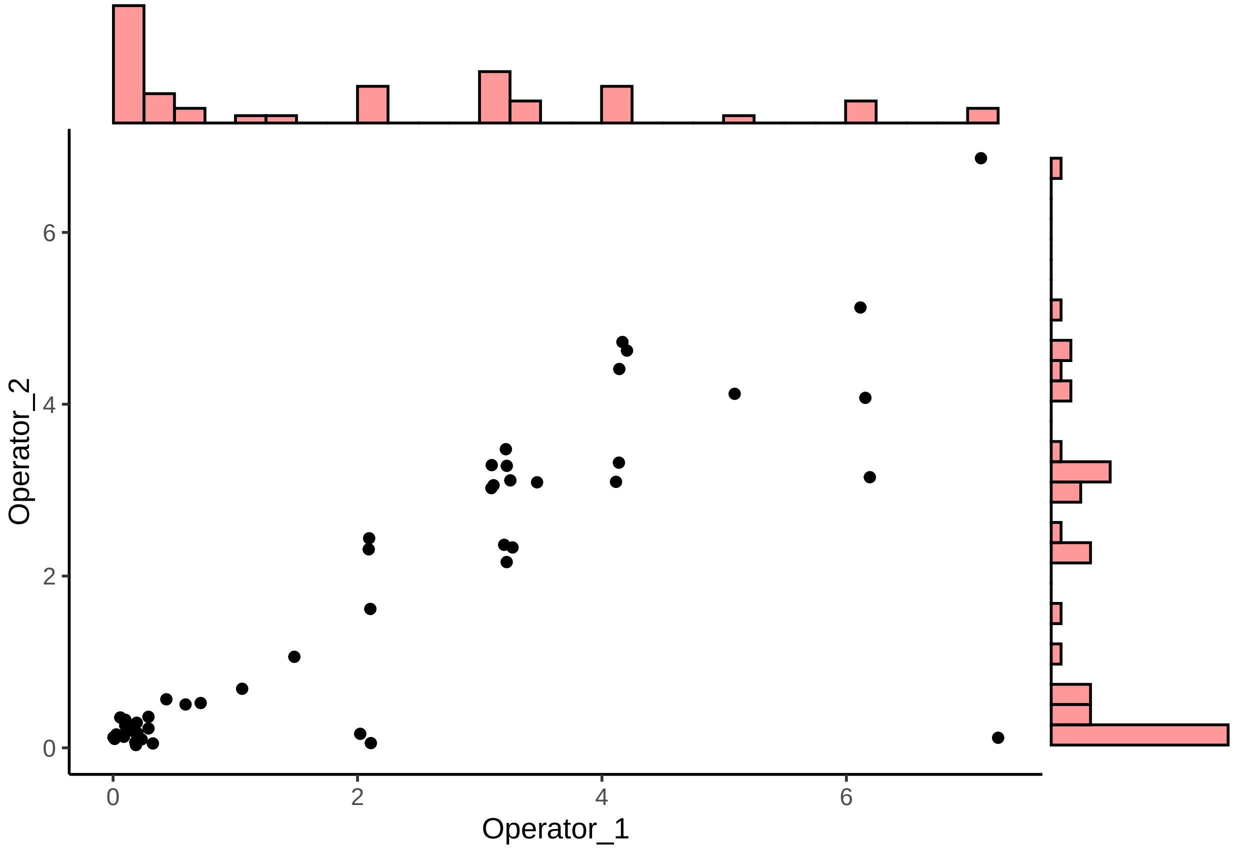

B. PEARSON AND SPEARMAN CORRELATION: FIGURE 1

The Figure 1 is plotting the scatter plot, linear regression line and value of Pearson values.

```
##################Figure 1 here...#######################################
#remove # SIGN in the next 2 lines to obtain the fig 1
#tiff("Fig_1.tiff", units="in", width=5, height=5, res=300)
ggplot(rel, aes(x=Rater_1, y=Rater_2))+geom_point()+
  geom_smooth(method="lm", se=FALSE, color="red",alpha=0.2)+
  stat_cor(label.y = 4, r.digits=3, color="red")+
  labs(x="Operator 1 (cm)", y="Operator 2 (cm)")+
  # annotate("text", x=1, y=6, color="black", label= expression("" ~rho ~ "=0.773, p<0.001"))+
     geom_abline(slope=1, intercept=0, color="blue", linetype="dashed", size=1)+
    annotate("text", x=5, y=6.2, color="blue", label= "Perfect fidelity y=x")+
  theme_classic()
```

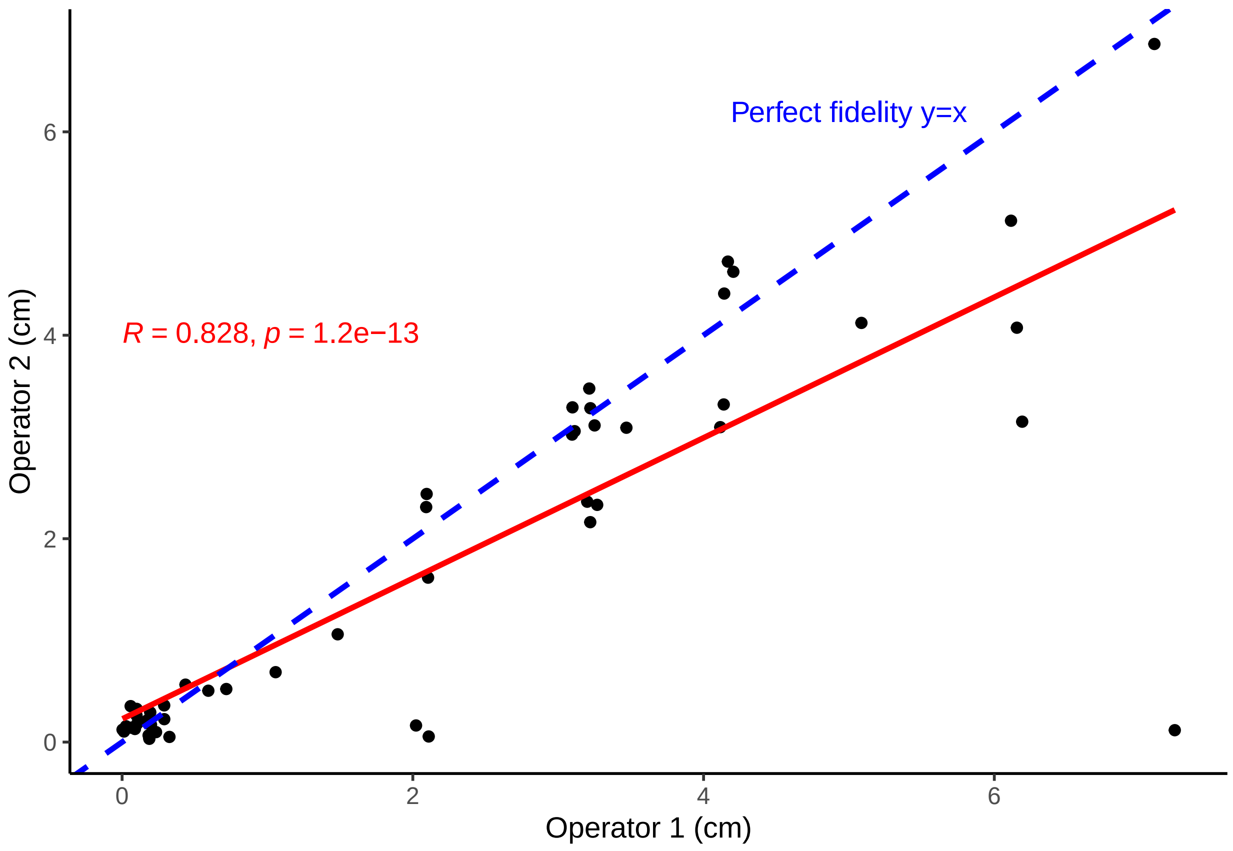


```
#dev.off()
```

The Spearman rho can be calculated simply by the following formula:

```
cor.test(x=rel$Rater_1, y=rel$Rater_2, method = 'spearman')
```

```
##
##  Spearman's rank correlation rho
```

```
##
## data:  rel$Rater_1 and rel$Rater_2
## S = 4720, p-value < 2.2e-16
## alternative hypothesis: true rho is not equal to 0
## sample estimates:
##       rho
## 0.7733493
```

The Spearman rho is 0.773.

We can also calculate the Lin's Concordance Correlation Coefficient (CCC) using the `CCC()` function of the `DescTools` package.

```
library(DescTools)
a <- CCC(rel$Rater_1, rel$Rater_2)
a$rho.c$est
```

```
## [1] 0.79404
```

The CCC value is 0.79404.

C. DEMING AND PASSING BABLOK REGRESSION: FIGURE 2

```
###################Figure 2 here...########################################
#remove # SIGN in the next 2 lines to obtain the fig 2
ex<- mcreg(rel$Rater_1, rel$Rater_2, method.reg = "PaBa") #obtaining Passing-Bablok regression
ex2<- mcreg(rel$Rater_1, rel$Rater_2, method.reg= "Deming") #obtaining Deming regression
ex3<- mcreg(rel$Rater_1, rel$Rater_2, method.reg= "WDeming")#obtaining Weighted Deming regression
```

```
## The global.sigma is calculated with Linnet's method
```

```
lin.reg <- lm(rel$Rater_2~rel$Rater_1) # obtaining linear regression
#tiff("Fig_2.tif", , width = 6, height = 6, units = 'in',res = 300)
plot(rel$Rater_1, rel$Rater_2, main = "", xlab = "Operator 1 (cm)", ylab = "Operator 2 (cm)", pch=16)
abline(ex@para[1:2], col = "black", lty=2)
abline(ex2@para[1:2], col = "black", lty=3)
abline(ex3@para[1:2], col = "black", lty=6)
abline(lin.reg, col="black",  lty=1)
legend("topleft", c("Passing-Bablok", "Deming", "Weighted Deming", "Linear regression"), lty=c(2,3,6,1)
```

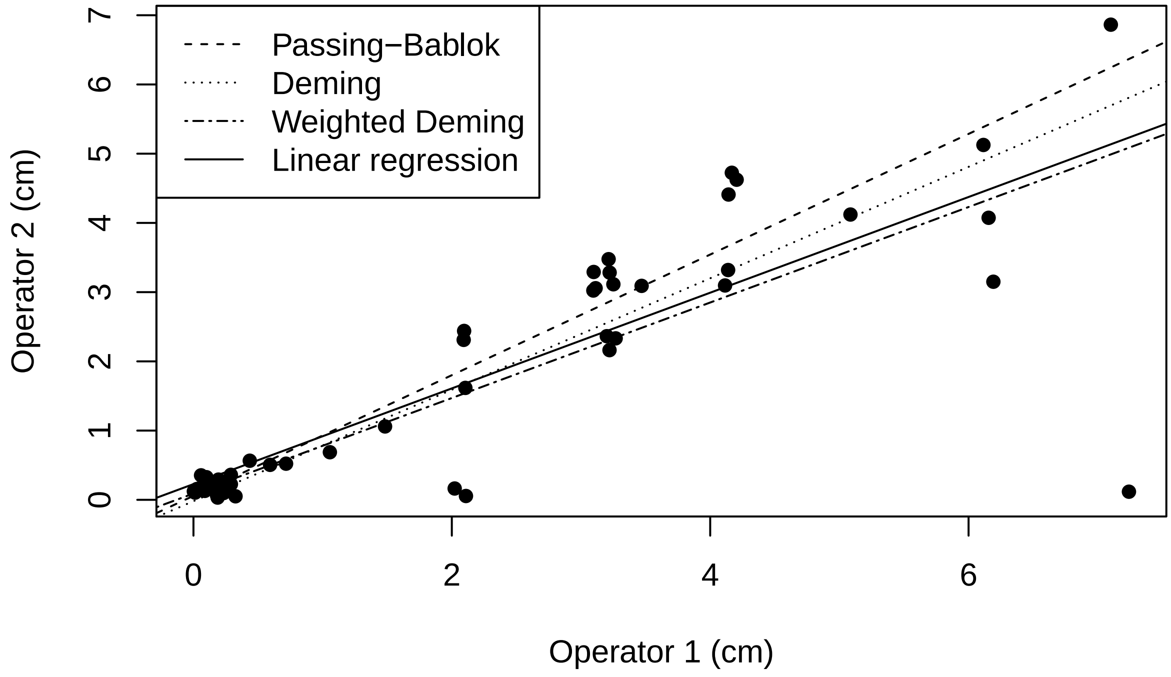


```
#dev.off()
```

D. AGREEMENT PLOT

D.1 Agreement plot (default Bland-Altman method): FIGURE 3

```
###################Figure 3 here...######################################
#remove # SIGN in the next 2 lines to obtain the fig 3

#pdf("Fig_3.pdf")
#tiff("Fig_3.tif", , width = 5, height = 4, units = 'in',res = 300)
blandr.draw(rel$Rater_1, rel$Rater_2)+theme_bw()+
  annotate("text", x=5.5, y=2.5, color="red", size=3, label= "Upper limit of agreement") +
  annotate("text", x=5.5, y=0.3,color="black",size=3,  fontface = "bold", label= "Mean bias") +
  annotate("text", x=5.5, y=-2.3, color="red",size=3,  label= "Lower limit of agreement")+
  ggtitle("")+
   ylab(expression(M[m1]-M[m2]))+
  xlab(expression((M[m1]+M[m2])/2))+
  theme_classic()
```

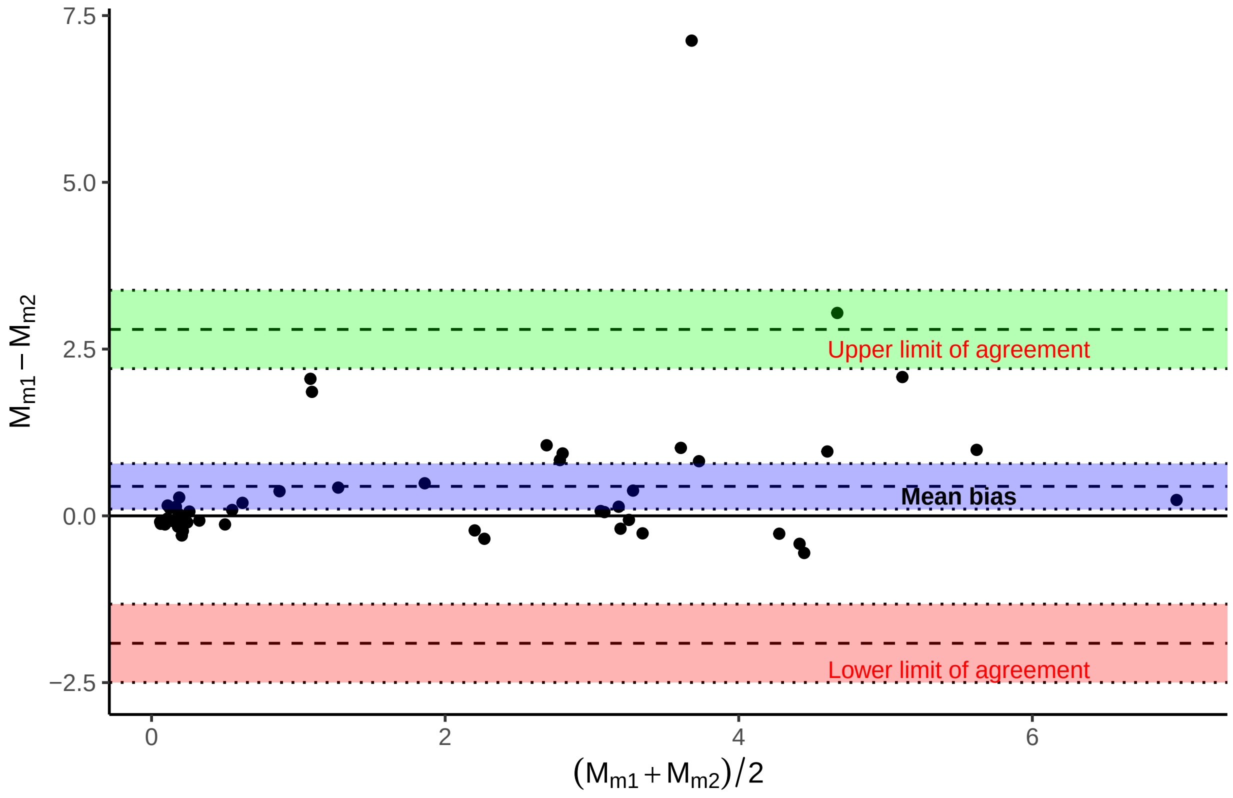

```
#dev.off()
```

D.2 Adjusted agreement plot allowing differential and proportional bias : FIGURE 4

```
##################Figure 4 here...########################################
#remove # SIGN in the next 2 lines to obtain the fig 4
rel$id <- c(1:50) #adding row id to identify the subject measured
rel$y2=rel$Rater_1
rel$y1=rel$Rater_2
#pdf("Fig_4.pdf")
bland_altman_plot(rel, new="y2",Ref="y1", ID="id", fill=TRUE)
```

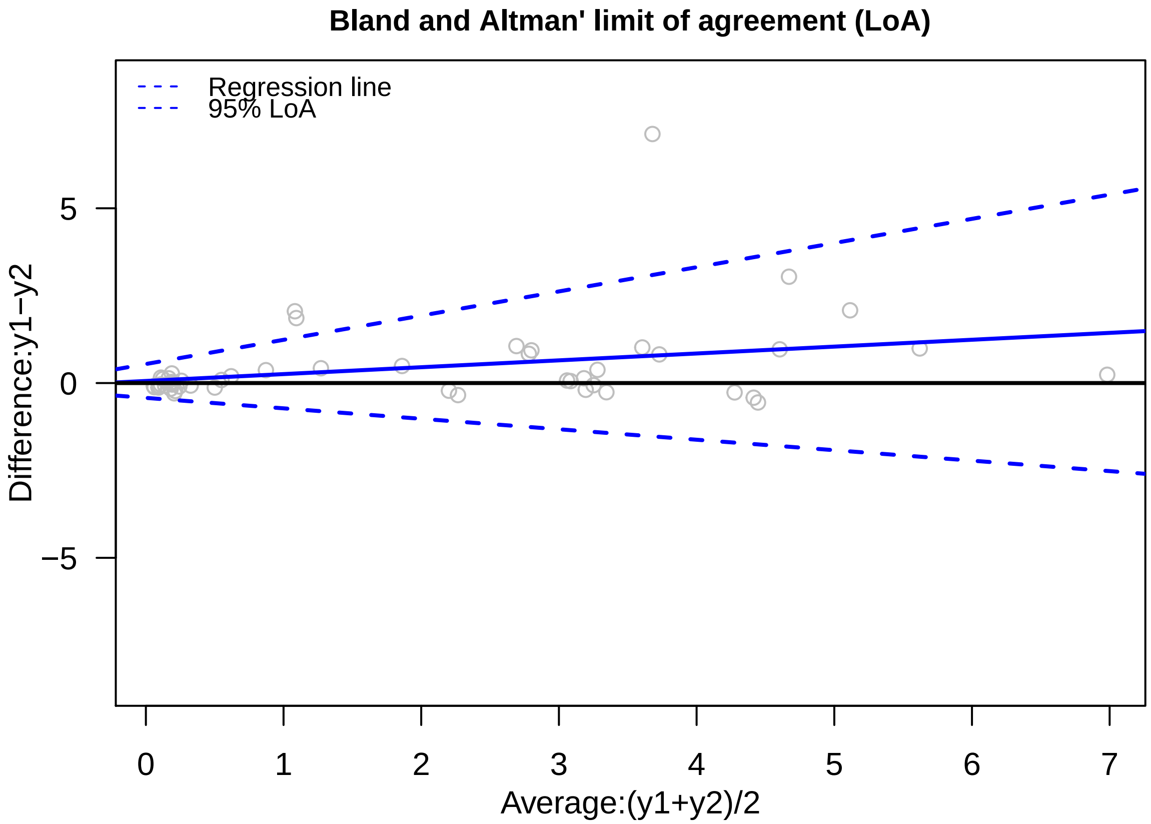


```
#dev.off()
```

ANY QUESTION=> s.buczinski@umontreal.ca
